# IT’S RAINING, IT’S POURING, THE OLD MAN IS SNORING: AGE STEREOTYPES IN NURSERY RHYMES

**DOI:** 10.1093/geroni/igae098.1139

**Published:** 2024-12-31

**Authors:** Reuben Ng, Nicole Indran

**Affiliations:** National University of Singapore, Singapore, Singapore; National University of Singapore, Singapore, Singapore

## Abstract

Ageist beliefs tend to take root in one’s formative years and persist into adulthood. As future older adults, children must be instilled with more positive attitudes towards aging. To gain insight into the ways in which children are being socialized to view old age, this study examines the depictions of old age in nursery rhymes. We built a comprehensive dataset of nursery rhymes by gathering material from four websites. In total, 735 unique rhymes were retrieved. To identify rhymes related to old age, we conducted a search using various age-related terms (e.g., old, elder, grandfather, grandma). This search yielded 85 nursery rhymes. After applying our exclusion criteria (e.g., instances where ‘old’ referred to buildings that have been around for a long time), we were left with 34 rhymes. Both inductive and deductive approaches guided our qualitative content analysis of the rhymes. Overall, old age was mentioned in 5% (N=34) of the 735 nursery rhymes. Of the 34 rhymes, half contained negative age stereotypes (50%; N=17). The remaining rhymes featured about an equal mix of positive (26%; N=9) and neutral age stereotypes (24%; N=9). In the context of an aging population, it is paramount that society is led by people with a realistic outlook of aging. Although nursery rhymes may seem like mere tales not to be taken seriously, they nonetheless remain powerful tools capable of molding thought processes. Our study sets the stage for telling children accurate and nuanced stories about older adults to inculcate healthy attitudes towards aging.

